# Extraskeletal Chondrosarcoma: Long-term Follow-up of a Patient with Metastatic Disease

**DOI:** 10.7759/cureus.2709

**Published:** 2018-05-30

**Authors:** Ahmed G Elsayed, Leesah Al-qawasmi, Heather Katz, Yehuda Lebowicz

**Affiliations:** 1 Hematology and Oncology, Joan C. Edwards School of Medicine at Marshall University, Huntington, USA

**Keywords:** extraskeletal chondrosarcoma, soft tissue sarcoma

## Abstract

Extraskeletal myxoid chondrosarcoma (EMC) is a rare soft tissue sarcoma with an indolent course and poor response to systemic treatment. We present a case of a 53-year-old male who presented with right gluteal extraskeletal myxoid chondrosarcoma. He was treated with wide local excision after receiving 50 Gray of neoadjuvant radiation therapy. Three years later he was found to have a left lower lobe lung nodule that was slowly increasing in size. He underwent a left lower lobectomy and the nodule was confirmed to be consistent with the patient’s history of EMC. One year later, lung imaging showed multiple small nodules bilaterally consistent with metastatic disease. The patient opted for watchful waiting approach. Routine follow-up imaging for four years shows a very slow progression of his disease burden. He continues to be asymptomatic. This case demonstrates the natural course of EMC and argues in favor of the watchful waiting approach in treating this disease.

## Introduction

Soft tissue sarcomas are rare tumors that originate from mesenchymal cells. Even though there are multiple distinct histological subtypes, they are usually lumped together in clinical trials to produce adequate sample size. Chondrosarcoma is a soft tissue sarcoma that is histologically characterized by the production of chondroid matrix. Extraskeletal myxoid chondrosarcoma (EMC) is a rare tumor characterized by cords or strands of small cells immersed in a myxoid matrix [1]. There are no clinical trials that investigated the best treatment options for this tumor given its very low incidence. Understanding of this tumor’s pathologic nature and clinical behavior comes mainly from case reports and retrospective reviews. The case presented is an example of the usual presentation of this rare tumor in a patient who opted not to receive systemic treatment, thus allowing us to see the natural course of this disease.

## Case presentation

**Figure 1:**
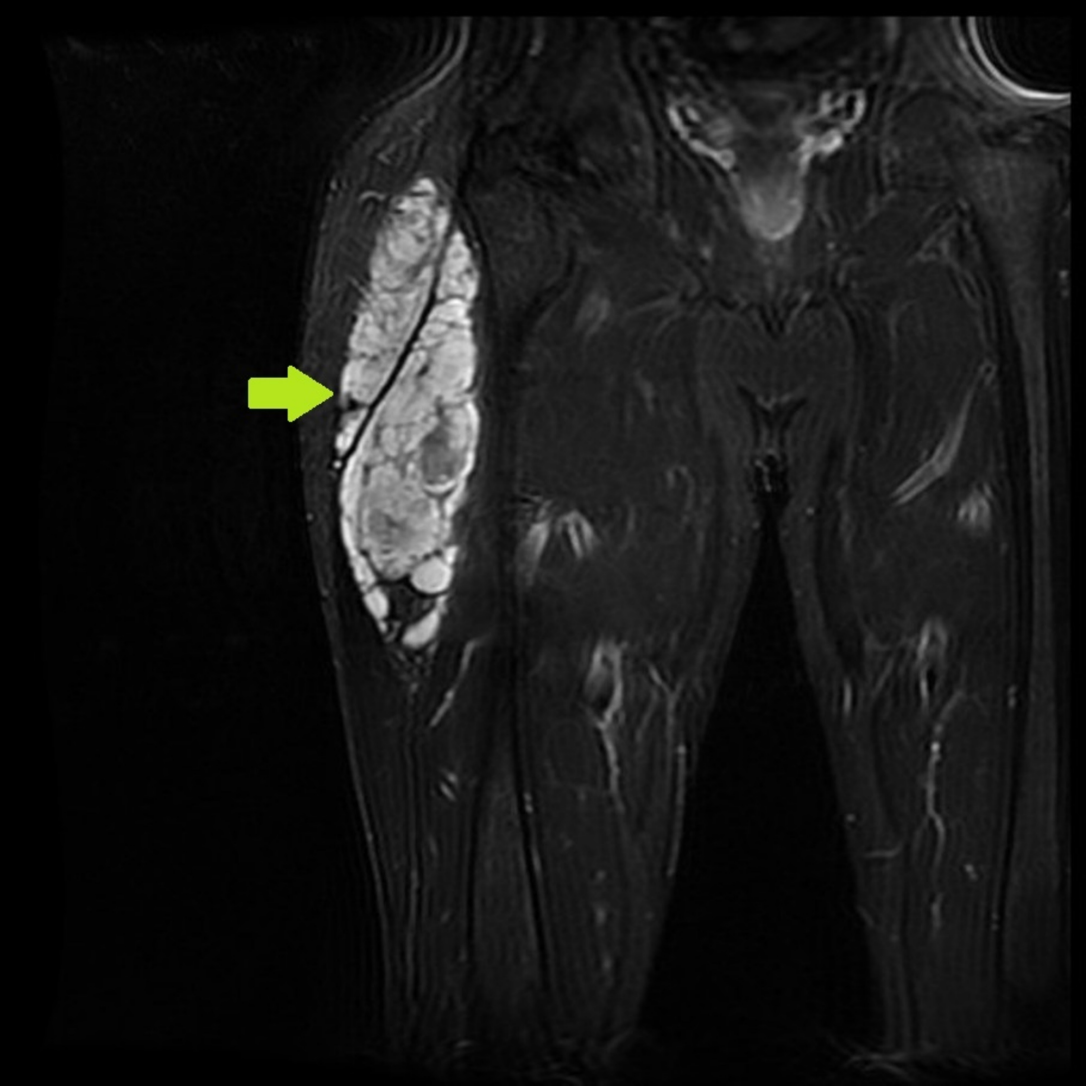
Magnetic resonance imaging (MRI) showing right gluteal extraskeletal myxoid chondrosarcoma.

A 53-year-old male presented in September of 2009 with a right gluteal mass (Figure 1).

**Figure 2:**
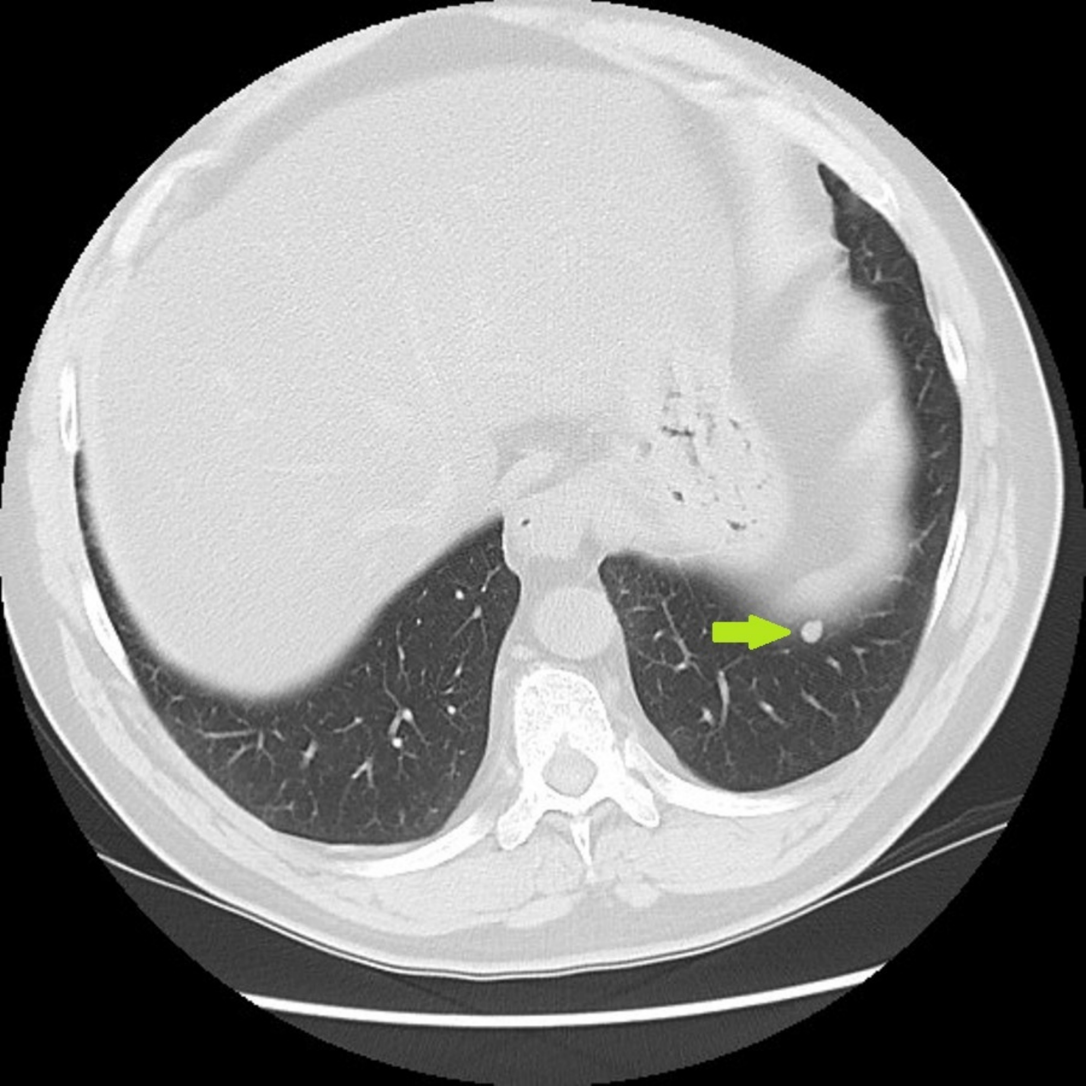
10/2012: One year after resection of primary tumor, computed tomography (CT) lung shows new left lower lung nodule.

Biopsy confirmed the presence of extraskeletal myxoid chondrosarcoma. He received neoadjuvant radiation treatment with total dose of 50 Gray, followed by wide local excision. In October of 2012, he was found to have a left lower lobe lung nodule (Figure 2) that was slowly increasing in size.

**Figure 3:**
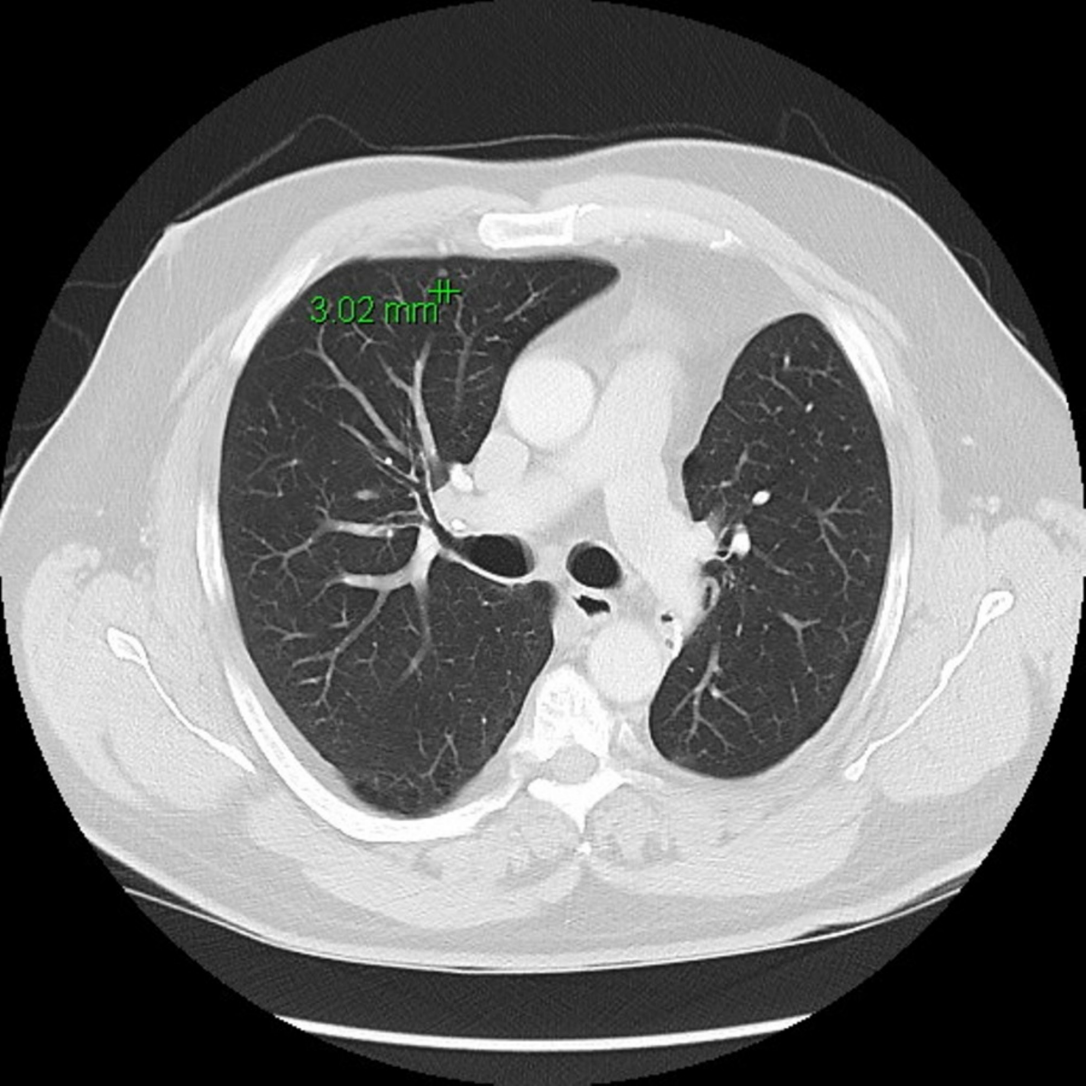
8/2013: New pulmonary nodules consistent with metastatic disease.

Because the nodule was close to the diaphragm, it could not be reached for biopsy. The patient underwent a left lower lobectomy. Pathology did confirm that the nature of this nodule was consistent with the patient’s history of EMC. He had surveillance imaging regularly afterward and was found to have multiple small pulmonary nodules in August of 2013 (Figure 3).

**Figure 4:**
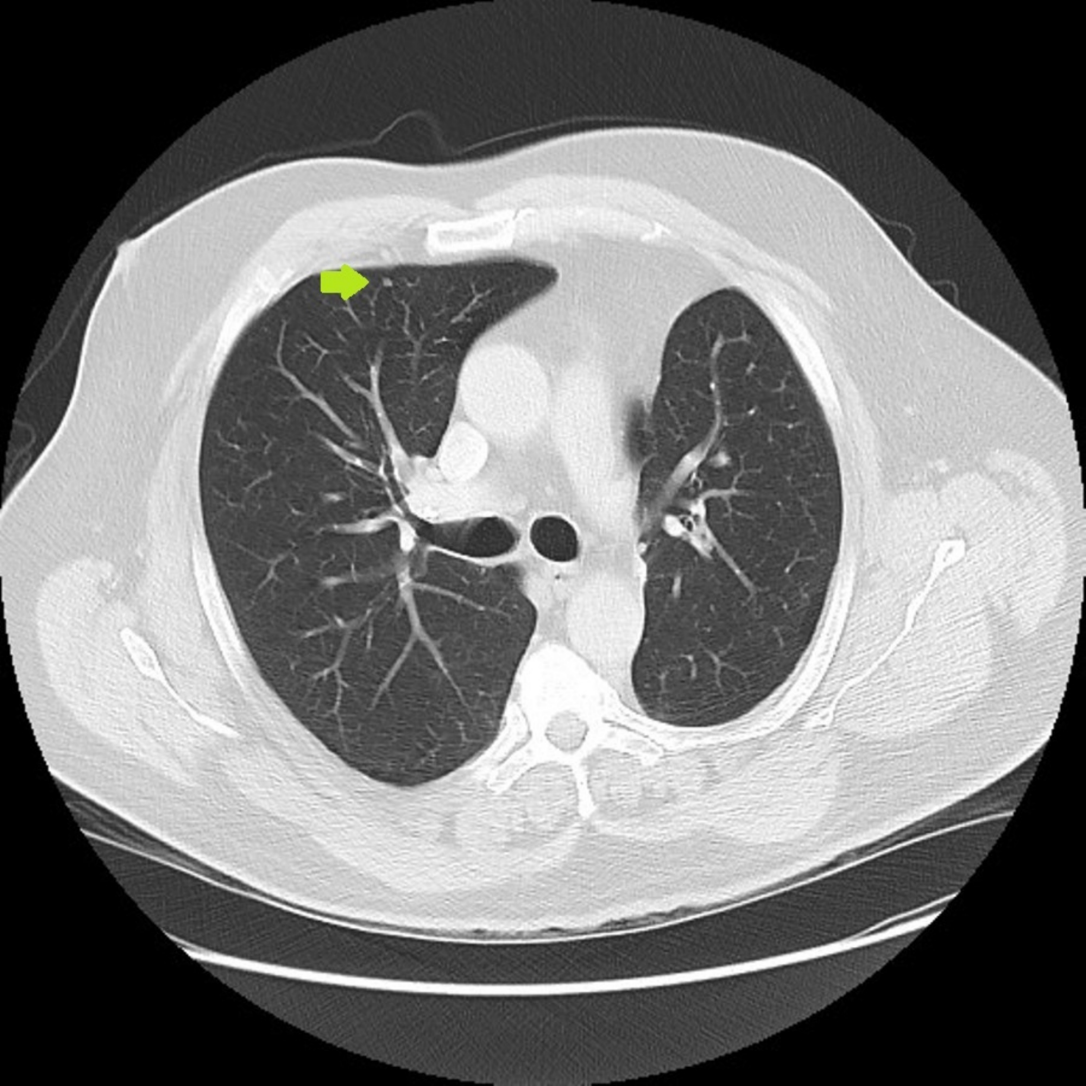
8/2014: Slow progression of lung nodules.

**Figure 5:**
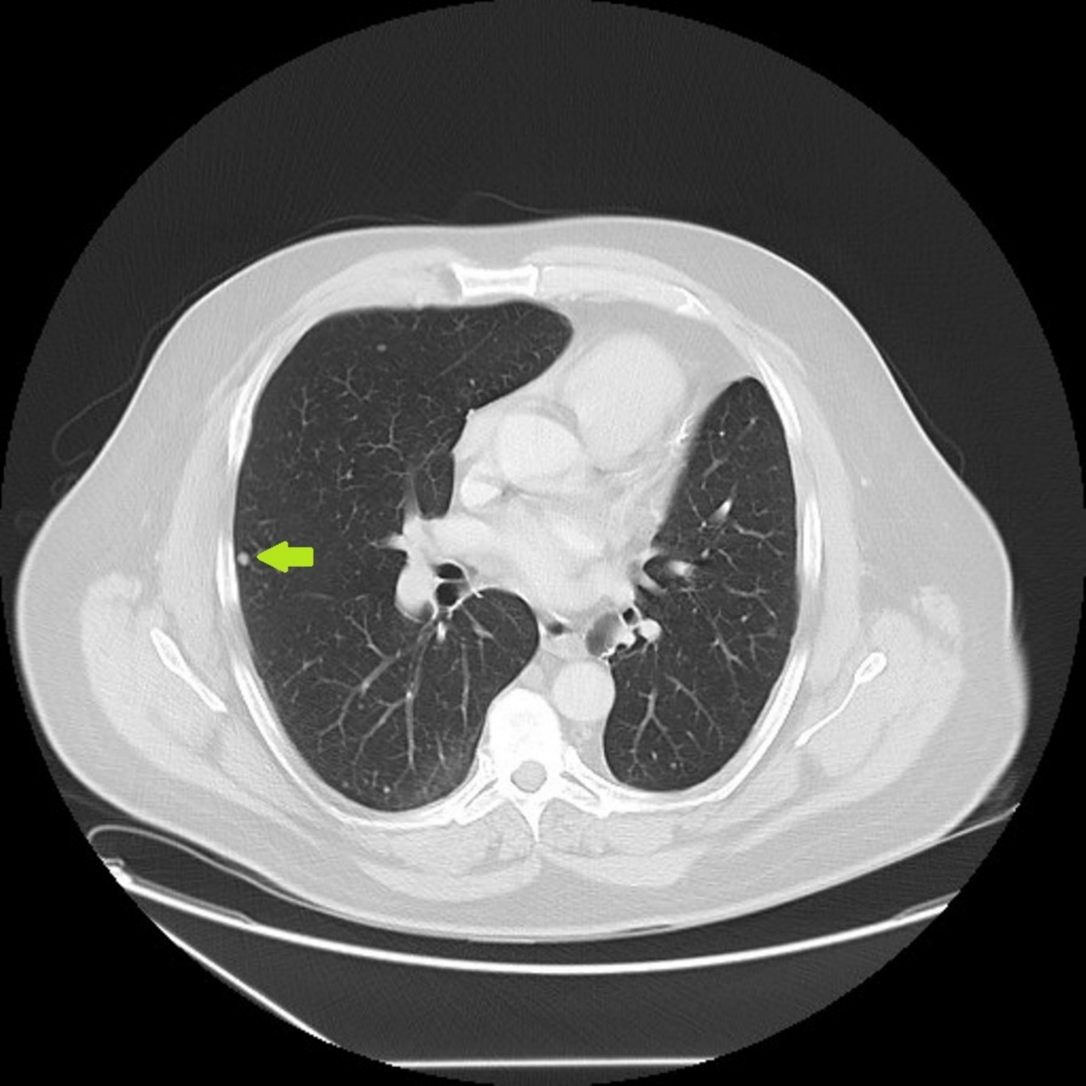
10/2015: Slow progression of lung nodules.

**Figure 6:**
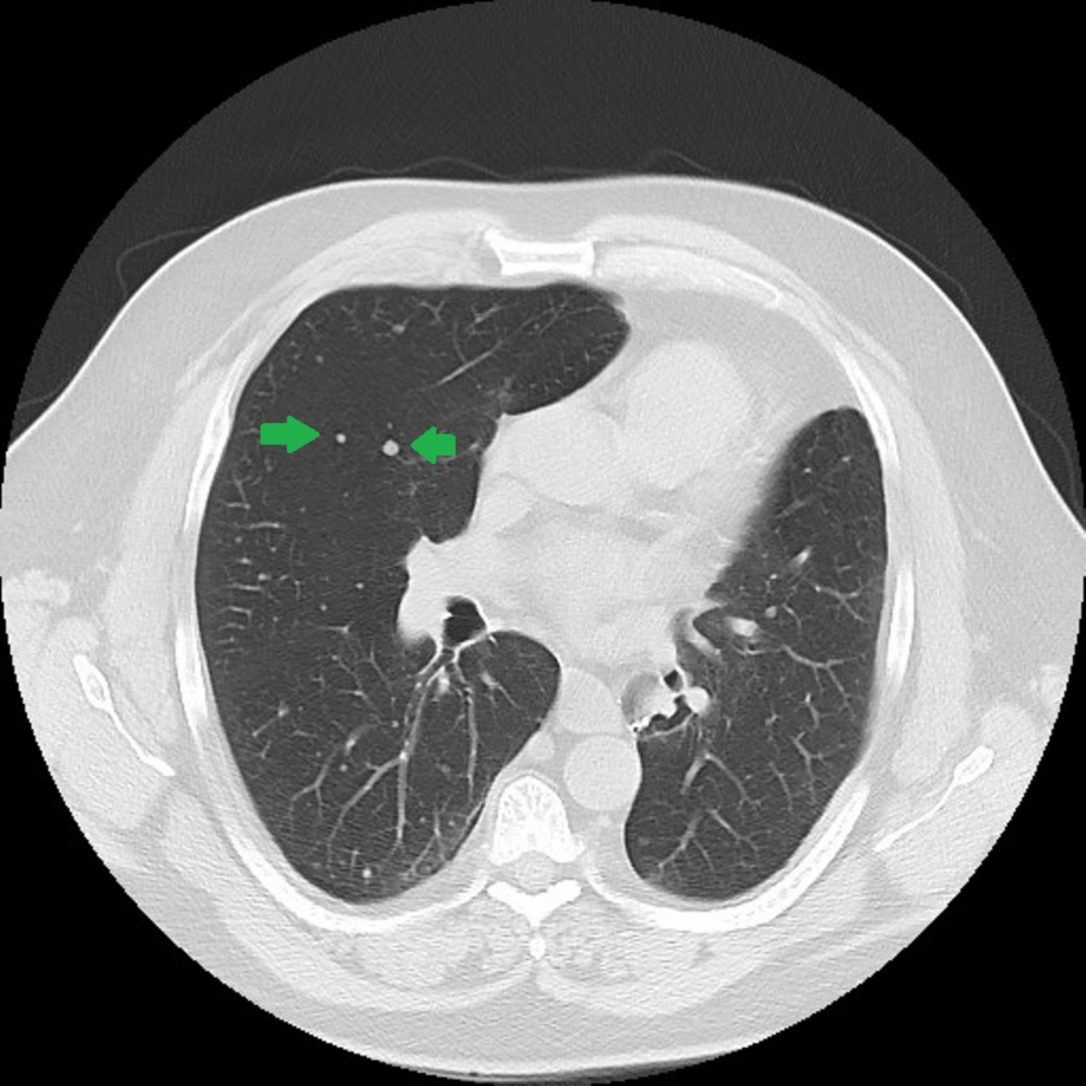
4/2016: Slow progression of lung nodules.

**Figure 7:**
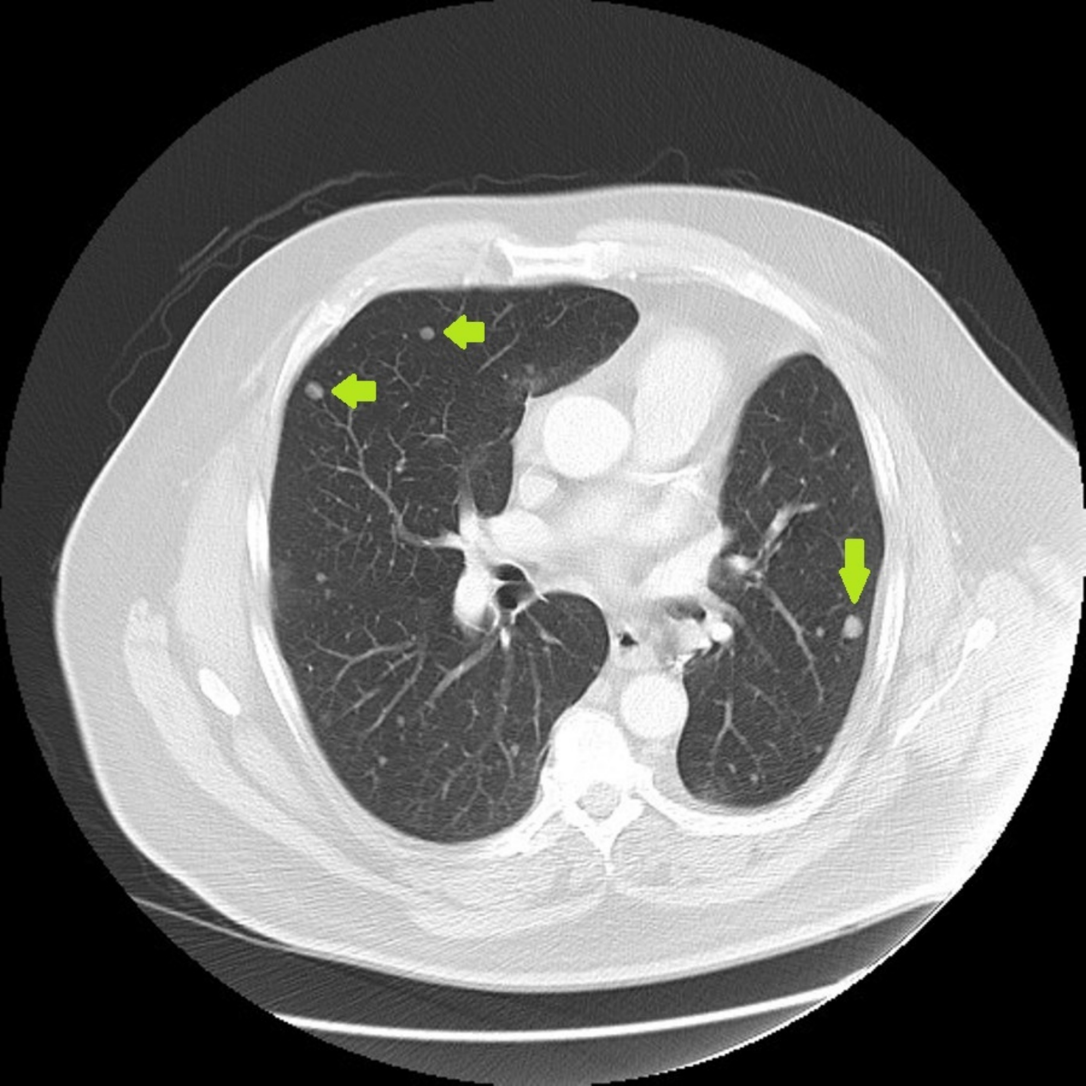
7/2017: Slow progression of lung nodules.

An extensive discussion and consultations with many experts were undertaken at that time. The patient opted for watchful waiting approach. Routine surveillance imaging since August of 2013 showed a progressive but slow increase of size of the multiple pulmonary nodules as well as the appearance of new nodules (Figures 4-7). Because the progression has been very slow, the patient remained asymptomatic.

## Discussion

EMC is a rare soft tissue sarcoma that was first recognized as a distinct entity in 1953 [2]. The disease usually presents during the fifth and sixth decades of life. The primary site is usually in the extremities although it can present in the abdomen and pelvis. There are case reports of other rare presentations such as lung, neck, mandible, orbit and heart [3-7]. More recently the tumor molecular characteristics were further established. The tumor harbors translocation (9;22) (q22;q11), resulting in EWSR1/NR4A3 sequence in the majority of patients. Other translocations have been described as well.

Localized disease is treated with wide local resection, while metastatic disease is often treated with systemic chemotherapy. Sarcoma regimens are usually used, such as Doxorubicin-based regimens or Ifosfamide-based regimens. Response to cytotoxic chemotherapy is usually poor. In one retrospective review, chemotherapy was able to produce stable disease for more than six months in 25% of patients [8]. Recent case series in the literature have reported decent treatment responses with sunitinib, but survival benefit at this time is unknown [9,10].

There is no strong evidence favoring a certain option in terms of systemic therapy. Given the indolent nature of this tumor and poor response to treatment, some experts favor the watchful waiting approach in treating metastatic disease after aggressive local management of primary tumor. The counter argument for this approach is that even though this is characteristically a slow growing tumor with late-onset metastases with 10-year survival ranging between 65 and 78%, aggressive variants have been described [11]. The presented case documents eight years follow-up of metastatic EMC that demonstrates the validity of the watchful waiting approach.

## Conclusions

Extraskeletal chondrosarcoma is a rare soft tissue sarcoma that usually presents in the extremities. Wide local resection can provide curative option in localized disease. It usually has an indolent course with poor response to systemic therapy, so watchful waiting is a valid approach in treating metastatic disease.
